# Artificial intelligence–generated apparent diffusion coefficient (AI-ADC) maps for prostate gland assessment: a multi-reader study

**DOI:** 10.1007/s00330-025-11871-z

**Published:** 2025-07-21

**Authors:** Kutsev Bengisu Ozyoruk, Stephanie A. Harmon, Enis C. Yilmaz, Erich P. Huang, David G. Gelikman, Sonia Gaur, Francesco Giganti, Yan Mee Law, Daniel J. Margolis, Pavan Kumar Jadda, Sitarama Raavi, Sandeep Gurram, Bradford J. Wood, Peter A. Pinto, Peter L. Choyke, Baris Turkbey

**Affiliations:** 1 Artificial Intelligence Resource, Molecular Imaging Branch, National Cancer Institute, Bethesda, MD USA; 2 Molecular Imaging Branch, National Cancer Institute, Bethesda, MD USA; 3 Division of Cancer Treatment and Diagnosis, National Cancer Institute, National Institutes of Health, Rockville, MD USA; 4 Department of Radiology, Brigham & Women’s Hospital, Boston, MA USA; 5 Department of Radiology, University College London Hospital NHS Foundation Trust, London, UK; 6 Division of Surgery & Interventional Science, University College London, London, UK; 7 Department of Radiology, Singapore General Hospital, Singapore, Singapore; 8 Weill Cornell Imaging, Cornell University, New York, NY USA; 9 Office of Information Technology, Center for Cancer Research, NCI, Bethesda, MD USA; 10 Urologic Oncology Branch, NCI, NIH, Bethesda, MD USA; 11 Center for Interventional Oncology, NIH Clinical Center, National Institutes of Health, Bethesda, MD USA; 12 Department of Radiology, Clinical Center, National Institutes of Health, Bethesda, MD USA

**Keywords:** Generative artificial intelligence, ADC map, Prostate, Magnetic resonance imaging

## Abstract

**Objective:**

To compare the quality of AI-ADC maps and standard ADC maps in a multi-reader study.

**Materials and methods:**

Multi-reader study included 74 consecutive patients (median age = 66 years, [IQR = 57.25–71.75 years]; median PSA = 4.30 ng/mL [IQR = 1.33–7.75 ng/mL]) with suspected or confirmed PCa, who underwent mpMRI between October 2023 and January 2024. The study was conducted in two rounds, separated by a 4-week wash-out period. In each round, four readers evaluated T2W-MRI and standard or AI-generated ADC (AI-ADC) maps. Fleiss’ kappa, quadratic-weighted Cohen’s kappa statistics were used to assess inter-reader agreement. Linear mixed effect models were employed to compare the quality evaluation of standard versus AI-ADC maps.

**Results:**

AI-ADC maps exhibited significantly enhanced imaging quality compared to standard ADC maps with higher ratings in windowing ease (β = 0.67 [95% CI 0.30–1.04], *p* < 0.05), prostate boundary delineation (β = 1.38 [95% CI 1.03–1.73], *p* < 0.001), reductions in distortion (β = 1.68 [95% CI 1.30–2.05], *p* < 0.001), noise (β = 0.56 [95% CI 0.24–0.88], *p* < 0.001). AI-ADC maps reduced reacquisition requirements for all readers (β = 2.23 [95% CI 1.69–2.76], *p* < 0.001), supporting potential workflow efficiency gains. No differences were observed between AI-ADC and standard ADC maps’ inter-reader agreement.

**Conclusion:**

Our multi-reader study demonstrated that AI-ADC maps improved prostate boundary delineation, had lower image noise, fewer distortions, and higher overall image quality compared to ADC maps.

**Key Points:**

***Question*** *Can we synthesize apparent diffusion coefficient (ADC) maps with AI to achieve higher quality maps?*

***Findings*** *On average, readers rated quality factors of AI-ADC maps higher than ADC maps in 34.80% of cases, compared to 5.07% for ADC (p < 0.01).*

***Clinical relevance*** *AI-ADC maps may serve as a reliable diagnostic support tool thanks to their high quality, particularly when the acquired ADC maps include artifacts.*

## Introduction

Multiparametric magnetic resonance imaging (mpMRI) has shown promising results in diagnosis, localization, risk stratification and staging of csPCa [1–5]. Based on prospective large-scale studies in the last decade, mpMRI has become a valuable technique for biopsy guidance and improves diagnostic yield of prostate biopsies [6–9], although some studies indicate mpMRI still misses 5–30% of PCa [10–12]. Successful use of mpMRI for biopsy guidance and PCa diagnosis is heavily dependent on image quality [13–16]. However, achieving high-quality MRI is challenging and requires adherence to acquisition protocols and consistent quality reporting, as specified in the Prostate Imaging Reporting and Data System (PI-RADSv2.1) guidelines [17–19]. Some factors, such as rectal gas-associated susceptibility artifacts and involuntary patient motion, can markedly degrade the image quality, subsequently lowering the diagnostic performance [20]. Diffusion imaging is heavily relied upon in the PI-RADSv2.1 diagnostic system, specifically the Apparent Diffusion Coefficient (ADC) map, but is prone to artifacts. Quality assurance initiatives such as Prostate Imaging Quality (PI-QUAL) are designed to improve image quality, yet the impact of these efforts on clinical practices may require a significant amount of time to become fully apparent [14, 21, 22]. Methods to improve the quality of ADC maps are, therefore, important for the diagnostic workflow.

Generative Adversarial Networks (GANs) [23] have demonstrated the ability to enhance image quality in medical imaging thanks to their reliance on simulated data distributions [24, 25], including in prostate applications [26–28]. In this study, we leverage a recently developed algorithm that synthesizes high-quality prostate organ-focused ADC maps from T2W MRI [28], where we aim to assess the quality of AI-ADC maps vs. standard ADC maps within a multi-reader study.

## Materials and methods

### Study design and population

Under an IRB-approved protocol (NCT03354416), we queried eligible patients who underwent mpMRI of the prostate consecutively between 10/01/2023 and 01/30/2024. All patients were scanned on a 3-Tesla MRI (Ingenia Elition X, Class IIa, Philips Healthcare) using a 32-channel phased array surface coil with reacquisition parameters given in Supplementary Table 1. All mpMRI scans were prospectively evaluated by an experienced genitourinary radiologist (B.T., with 17 years of experience and 1000 prostate scan evaluations per year) using PI-RADS v2.1 and PI-QUAL v1 [14].

**Fig. 1:**
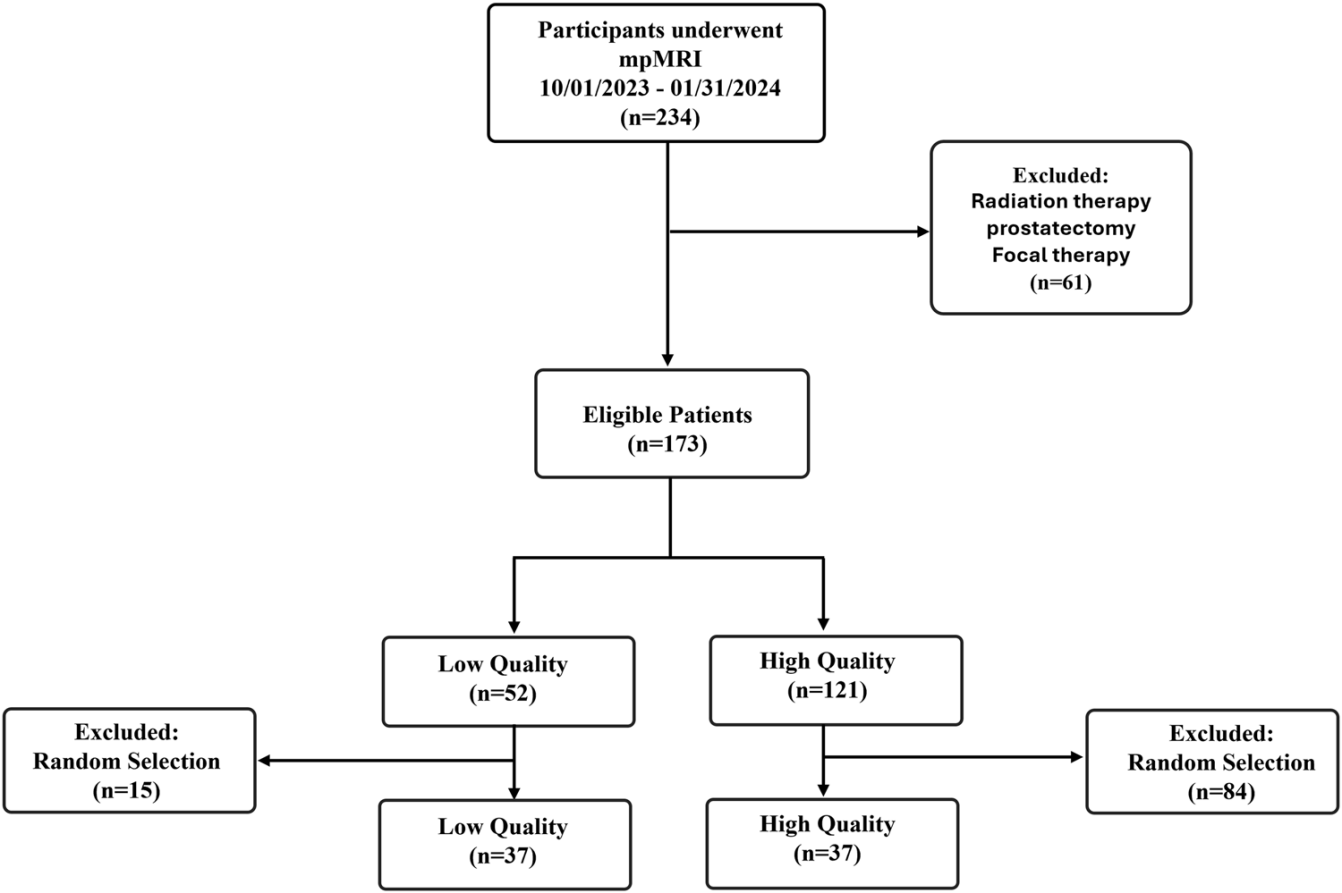
Study flowchart. Study inclusion and exclusion. mpMRI, multiparametric MRI

Random selection was applied to retrospectively construct a study population of 74 patients (Fig. 1) based on power analysis for inter-reader kappa statistics [29, 30] with an equal representation of low and high quality MR images, assuming a baseline kappa value of 0.2 for ADC maps, a target kappa value of 0.4 for AI-ADC maps, and α = 0.05 to achieve a power of 0.9.

### Generation of AI-ADC maps

AI-ADC [28] methodology was used to generate ADC maps from T2W MR images, which will be referred to as “AI-ADC” maps throughout the remainder of this study. Briefly, the axial T2W MRI is cropped using the prostate organ boundary obtained by a prostate segmentation AI model [31]. Following the cropping procedure, the AI-ADC model synthesizes ADC maps using contrastive learning and spatial and channel attention mechanisms without utilizing ADC maps from acquired DWI [28] (https://github.com/NIH-MIP/AI-ADC).

### Multi-reader study

**Fig. 2:**
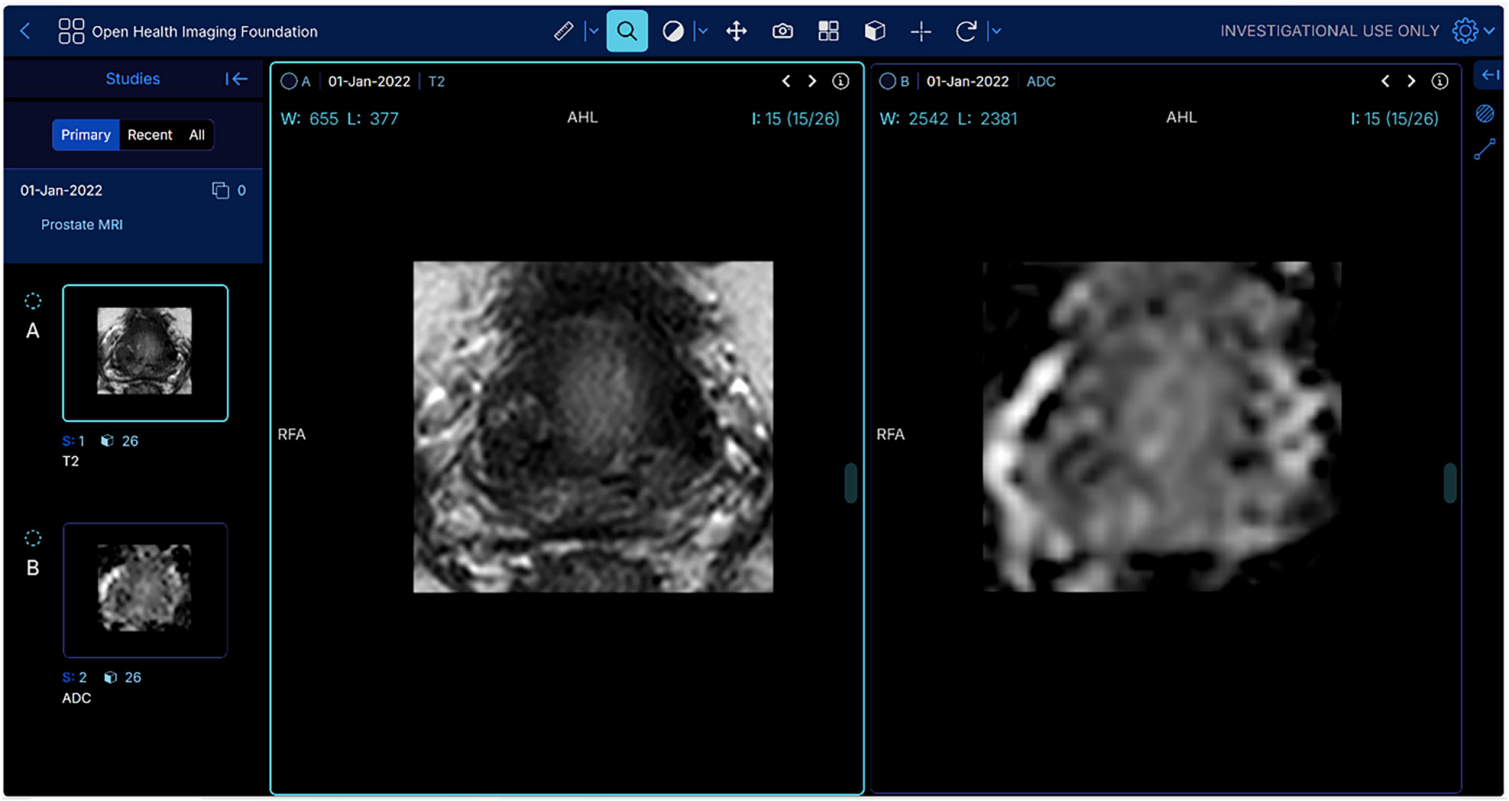
Prostate-focused T2W MRI and ADC maps. The readers evaluated 74 pairs of T2W MRI and ADC maps using side-by-side visualization in the displayed format. They had the option to adjust the windowing settings and could reset the view to explore different viewing preferences if needed. A zoom option was also available

We included four board-certified radiologists from four different institutions, each with experience reading over 300 prostate MRI scans per year. Readers were blinded to the study objective, clinical, and histopathological outcomes. A multi-reader study was conducted in two rounds, separated by a 4-week wash-out period, where readers evaluated T2W MRI and ADC map pairs (Fig. 2) by answering 12 questions on image quality while blinded to the ADC map type (standard ADC vs. AI-ADC). In the second round, readers were shown the opposite combination of T2W MRI and ADC pairs (i.e., round 1 T2 + ADC and round 2 T2 + AI-ADC). Combinations were randomly assigned and ordered across both rounds to avoid memory recall bias. The full list of questions and the graphical user interface details are provided in the Supplementary Material.

### Statistical analysis

Reader responses were considered as ordinal data for statistical analysis. The frequency (proportion) of cases where AI-ADC was scored higher than ADC (or vice versa) was recorded. Statistical evaluation was performed using the Wald test based on 1000 bootstrap iterations on the patient level for each reader individually and the average of all readers.

Linear mixed effects models were used to evaluate the effect of modality (AI-ADC vs. ADC) on quality ratings, adjusting for random effects of case and reader. A generalized linear mixed model (GLMM) with a binomial output was used for binary questions and a cumulative link mixed model (CLMM) with Laplace approximation for multinomial questions. At the reader level, the Wilcoxon signed-rank test with continuity correction of paired differences in scoring was reported.

Quadratic-weighted Cohen’s kappa scores were calculated for each pair of readers [32]. The overall agreement level of four readers was assessed by calculating Fleiss’ kappa Score [33, 34]. All statistical testing was performed using R (2023.09.1). For all evaluations, *p* < 0.05 was considered significant, and for bootstrap procedures, 95% confidence intervals are reported.

## Results

Of the 12 study questions, our primary objective focused on 7 questions which were related to image quality, including: windowing ease, prostate boundary delineation, distortion levels, noisiness, overall quality assessment and whether or not repeat acquisition of the sequence would be required during routine clinical assessment. Windowing, prostate boundary assessment, distortion, and level of image noise were all ranked using a 0–3 scale, with 3 reflecting the highest/most favorable imaging characteristics. All other questions were ranked using a binary scale, with 1 being the highest/most preferred characteristic.

### Qualitative comparison of ADC maps vs. AI-ADC maps

**Fig. 3:**
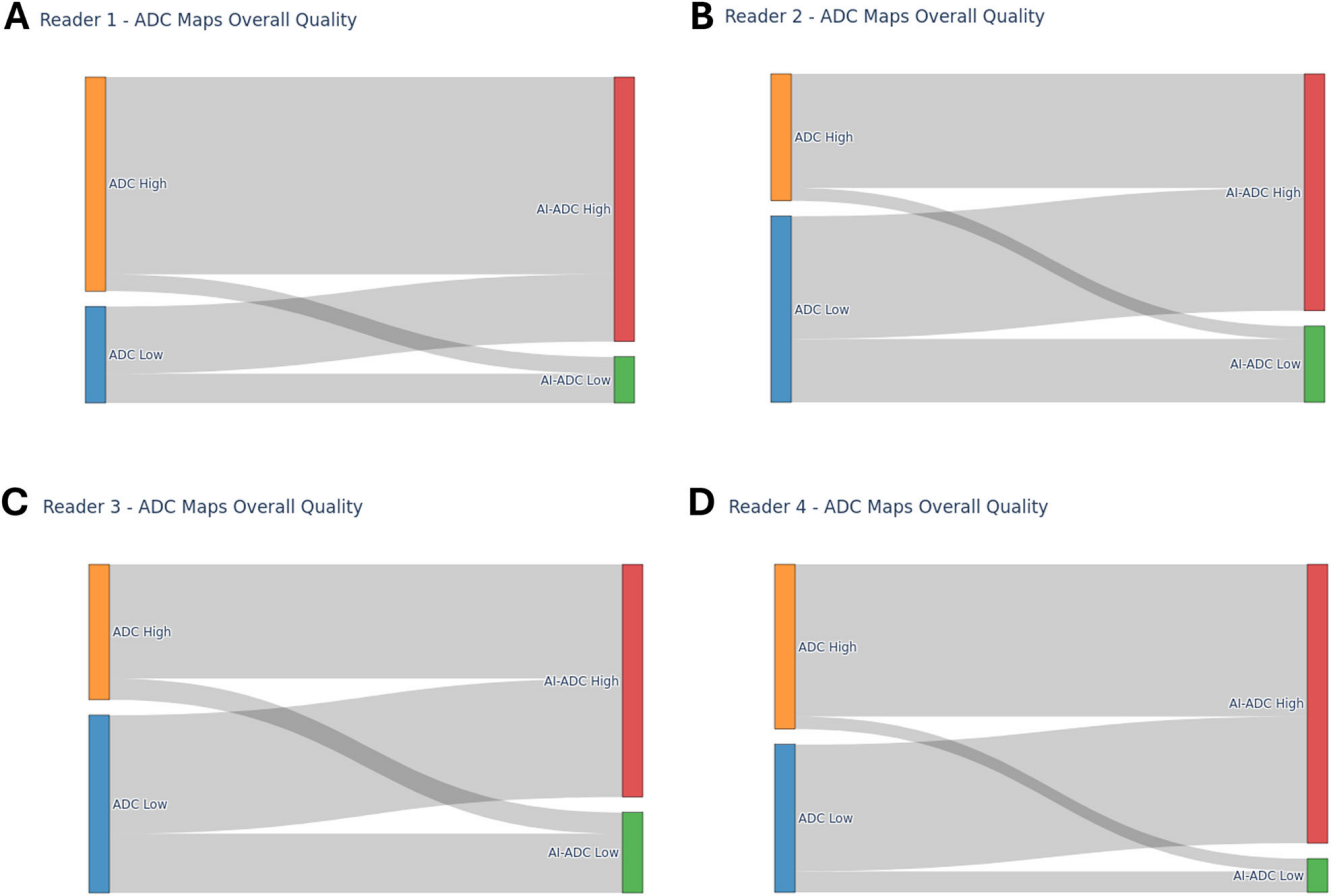
Sankey diagrams for overall ADC map quality evaluations. Reader 1 evaluated 68% (50/74) of ADC maps as being of high quality, whereas 85% (63/74) of AI-ADC maps were evaluated as being of high quality (**A**). Reader 2 evaluated 41% (30/74) of ADC maps as being of high quality, whereas 77% (56/74) of AI-ADC maps were evaluated as being of high quality (**B**). Reader 3 evaluated 43% (32/74) of ADC maps as being of high quality, whereas 74% (55/74) of AI-ADC maps were evaluated as being of high quality (**C**). Reader 4 evaluated 53% (39/74) of ADC maps as being of high quality, whereas 89% (66/74) of AI-ADC maps were evaluated as being of high quality (**D**)

**Fig. 4:**
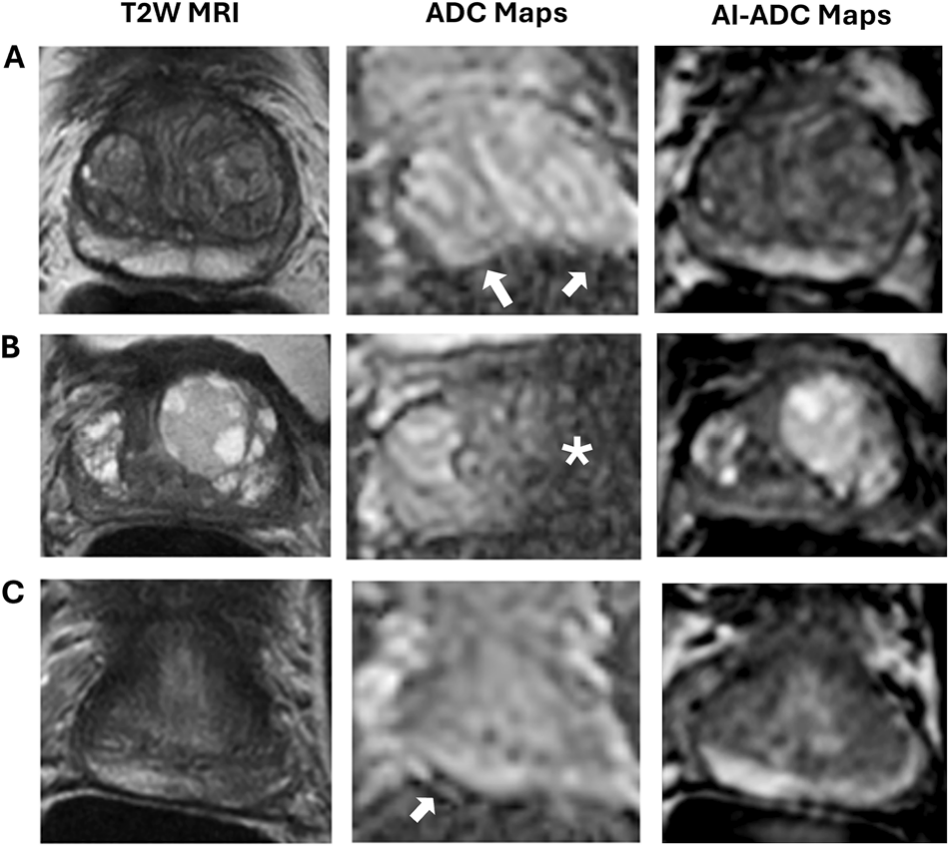
Examples of three patients with T2W MRI, ADC maps, and AI-ADC maps. An 80-year-old patient with a serum PSA of 10.3 ng/mL presented with a standard ADC map distorted due to rectal gas-associated susceptibility artifacts (arrows) with a concave boundary artifact in the posterior prostate; however, the AI-ADC map generated from T2W MRI showed no artifact (**A**). An 83-year-old patient with serum PSA of 1.8 ng/mL had a standard ADC map with significant noise in the left hemi-prostate secondary to a left hip prosthesis (asterix), but the AI-ADC map did not demonstrate this noise (**B**). A 57-year-old patient with serum PSA of 0.7 ng/mL had a standard ADC map affected by rectal gas susceptibility artifacts (arrow), with the corresponding AI-ADC map remaining artifact-free (**C**). All readers gave higher quality scores for AI-ADC maps for these three patients

Overall, the proportion of scans where AI-ADC achieved higher scores than its corresponding ADC was statistically significant for all categories related to diffusion imaging (Table 1). The categories with the highest differential in scoring were distortion (AI-ADC > ADC in 44.93% of cases vs. ADC > AI-ADC in 11.49% of cases, *p* < 0.001) and overall image quality (AI-ADC > ADC in 34.80% of cases vs. ADC > AI-ADC in 5.07% of cases, *p* < 0.001). Sankey diagrams for overall quality evaluations of ADC and AI-ADC maps per reader are presented in Fig. 3, and sample cases where AI-ADC maps received higher quality scores are presented in Fig. 4 (Supplementary Fig. 1). The per-reader analyses were generally consistent with the average reader evaluations (Supplementary Table 2). Notably, all four readers agreed on a higher overall quality of AI-ADC maps (all *p* < 0.01), as well as a reduced need for image reacquisition with AI-ADC maps (all *p* < 0.01).

**Table 1:** Average proportion of cases (*N* = 74) rated higher for AI-ADC maps versus ADC maps by readers and vice versa

(Criteria/all readers)	AI-ADC > ADC	ADC > AI-ADC	Difference	*p*-value
Windowing easiness	27.70% [0.230, 0.331]	15.54% [0.111, 0.199]	12.16% [0.044, 0.206],	*p* < 0.05
Prostate boundary delineation	43.24% [0.365, 0.503]	14.19% [0.098, 0.193]	29.05% [0.186, 0.399]	*p* < 0.001
Distortion evaluation	44.93% [0.368, 0.527]	11.49% [0.081, 0.155]	33.44% [0.230, 0.429]	*p* < 0.001
Noise evaluation	30.07% [0.257, 0.348]	15.20% [0.111, 0.193]	18.82% [0.088, 0.216]	*p* < 0.001
Overall quality evaluation of T2W MRI	7.43% [0.044, 0.108]	5.74% [0.034, 0.081]	1.25% [−0.024, 0.057]	*p* > 0.05
Overall quality evaluation of ADC maps	34.80% [0.270, 0.432]	5.07% [0.030, 0.074]	29.73% [0.203, 0.392]	*p* < 0.001
Image reacquisition requirements for ADC maps	29.73% [0.226, 0.361]	4.05% [0.020, 0.068]	25.68% [0.176, 0.335]	*p* < 0.001

To more directly compare the ordinal scoring of AI-ADC and ADC, linear mixed effects models were used to evaluate differences in scoring after adjusting for variability due to readers and patients (Table 2). Image distortion had the highest increase in scores between ADC and AI-ADC (mean score 2.53 [95% CI 2.44–2.60] and 1.98 [95% CI 1.87–2.09], respectively) and estimated coefficient (β) from the CLMM for AI-ADC maps was 1.68 (95% CI: 1.30–2.05, *p* < 0.001), indicating that AI-ADC maps were associated with significantly higher quality scores compared to ADC maps. The highest β effect sizes were observed in the overall quality evaluation of ADC map (2.21 [95% CI 1.59–2.67], *p* < 0.0001) and image reacquisition requirement (2.23 [1.69–2.76], *p* < 0.001), again reflecting a significant improvement in scoring between ADC and AI-ADC. Cumulative reader score evaluations were consistent with the per-reader quality assessments (Supplementary Table 3).

**Table 2:** Average scoring on ADC and AI-ADC maps across four readers

Criteria (score range)	ADC scores mean [95% CI]	AI-ADC scores mean [95% CI]	β [95% CI]	*p*-values
Windowing easiness (0–3)	2.00 [1.89–2.11]	2.18 [2.08–2.26]	0.67 [0.30–1.04]	*p* < 0.05
Prostate boundary delineation (0–3)	1.63 [1.53–1.72]	2.05 [1.96–2.14]	1.38 [1.03–1.73]	*p* < 0.001
Distortion evaluation (0–3)	1.98 [1.87–2.09]	2.52 [2.44–2.60]	1.68 [1.30–2.05]	*p* < 0.001
Noise evaluation (0–3)	1.59 [1.50–1.68]	1.87 [1.76–1.98]	0.56 [0.24–0.88]	*p* < 0.001
Overall quality evaluation of T2W MRI (0–1)	0.81 [0.77–0.86]	0.83 [0.78–0.87]	0.23 [−0.37, 0.82]	*p* > 0.05
Overall quality evaluation of ADC maps (0–1)	0.51 [0.46–0.57]	0.81 [0.77–0.86]	2.21 [1.59–2.67]	*p* < 0.0001
Image reacquisition requirements for ADC maps (0–1)	0.60 [0.55–0.66]	0.86 [0.82–0.90]	2.23 [1.69–2.76]	*p* < 0.001

Results of generalized linear mixed effects and cumulative link mixed model for which the scores are calculated as ADC_scores ~ modality (ADC or AI-ADC maps) + (1 | reader) + (1 | case)

### Inter-reader agreement

Overall inter-reader agreement was broadly poor-to-moderate for quality-related survey questions (Table 3). A statistically significant increase in QWK was observed for distortion evaluation (κ = 0.042 [95% CI −0.06 to 0.136] with ADC vs. κ = 0.175 [95% CI 0.065–0.296] with AI-ADC, *p* < 0.05). Pairwise inter-reader agreement across six reader pairs (Supplementary Table 4) and overall agreement among the four readers (Supplementary Table 5) demonstrated fair agreement regarding the superiority of AI-ADC maps over ADC maps in terms of overall quality evaluations.

**Table 3:** Mean quadratic-weighted kappa on ADC and AI-ADC maps across four readers

Criteria (score range)	QWK ADC [95% CI]	QWK AI-ADC [95% CI]	*p*-values
Windowing easiness (0–3)	−0.008 [−0.067, 0.044]	0.034 [−0.024, 0.121]	*p* > 0.05
Prostate boundary delineation (0–3)	0.287 [0.222, 0.348]	0.310 [0.191, 0.435]	*p* > 0.05
Distortion evaluation (0–3)	0.042 [−0.064, 0.136]	0.175 [0.065, 0.296]	*p* < 0.05
Noise evaluation (0–3)	−0.014 [−0.051, 0.021]	0.085 [0.032, 0.146]	*p* < 0.05
Overall quality evaluation of T2W MRI (0–1)	0.355 [0.296, 0.418]	0.284 [0.179, 0.387]	*p* > 0.05
Overall quality evaluation of ADC maps (0–1)	0.443 [0.381, 0.487]	0.438 [0.384, 0.500]	*p* > 0.05
Image reacquisition requirements for ADC maps (0–1)	0.365 [0.280, 0.449]	0.234 [0.090, 0.367]	*p* < 0.05

## Discussion

In this study, the potential of AI-ADC maps to enhance prostate gland assessment was evaluated with four readers blinded to whether they were reading AI-ADC or ADC maps [35, 36]. AI-ADC maps were entirely derived from the T2W MRI, whereas ADC maps were directly calculated from the acquired DW MRI. Our multi-reader analysis demonstrated that readers preferred AI-ADC maps over ADC maps due to their higher quality. Here, readers indicated that AI-ADC maps achieved higher scores in their ability to delineate prostate boundary with less noise and distortion and determine overall quality, resulting in fewer recommendations for repeat acquisitions. The estimated β coefficients from generalized linear mixed models and cumulative link mixed models reflect the magnitude of improvement offered by AI-ADC maps, where the size of the β values indicates the strength of the quality difference between standard ADC maps vs. AI-ADC maps: higher β values correspond to more pronounced improvements in image quality and usability. Among the evaluated domains, major factors in image quality benefited the most, including reduction in reacquisition preference, image enhancement, and presence of distortion. A substantial reduction in the need for repeat imaging was noted, directly translating to improved workflow efficiency and reduced patient burden.

Were AI-ADC to be adopted, the mpMRI protocol could potentially be shortened, possibly even to a single sequence, while also reducing the likelihood of reacquisition. This would lead to more efficient clinical workflows, minimizing repeat scans and optimizing resource allocation in high-volume clinical settings. Recent literature has shown the potential clinical value of generative AI models for image synthesis. Podgorsak et al [37] generated synthetic MR images from CT data using a PCGAN model to streamline prostate high-dose-rate brachytherapy treatment planning. Observer performance in delineating prostate boundaries showed that synthetic MRIs were accurate enough to produce treatment plans comparable to those derived from real MRIs [37]. A recent study [38] further demonstrated the feasibility of generating simulated contrast-enhanced prostate MRI from non-contrast sequences (T1W, T2W, DWI, and ADC maps). Using a pix2pix model, simulated contrast-enhanced images had high similarity to acquired images, showed excellent inter-reader agreement (Cohen κ = 0.96; 95% CI: 0.94–0.98), and demonstrated clinical relevance based on reclassification of PI-RADS version 2.1 scoring [38]. While our methodology and purpose differ in this study, we similarly observe that synthetic images derived from anatomical sequences result in images that have higher anatomical definition (organ boundary distinction) and improved levels of noisiness and distortion, ease of windowing, and overall quality scores of AI-ADC.

Previous studies have highlighted substantial inter-reader variability in the evaluation of DWI features within prostate MRI, underscoring a critical challenge in consistent image interpretation. In Giganti et al [39], strong reproducibility in PI-QUAL assessment between two radiologists was reported overall. However, in cases with notable disagreement, the greatest discrepancies emerged in the evaluation of the DWI sequence on the basis of overall image adequacy and absence of artifacts. Similarly, in Basar et al [40], report high inter-reader agreement in diagnostic quality of T2W (κ = 0.76 [95% CI: 0.56–0.96]) compared to DWI (κ = 0.46 [95% CI: 0.18–0.74]). In our study, despite the demonstrated improvements in ADC maps’ image quality, we did not observe a consistent trend toward higher inter-reader agreement for AI-ADC maps compared to standard ADC maps.

Our study has some limitations. First, our sample size of 74 patients, although sufficient in terms of power to detect a clinically relevant difference in agreement levels, was relatively small. Second, the scope of the study was limited by the diversity of the dataset used. Although the ADC maps obtained from one institute exhibit some diversity due to the inclusion of both high- and low-quality groups in the test set, incorporating more diverse datasets from different MRI systems and institutions would further ensure the robustness of the AI-ADC model across varied clinical environments. Future studies will consider multi-center trials to validate the model’s performance on different scanners, acquisition protocols, and patient populations. Third, we only evaluated the quality and utility of AI-ADC maps in a retrospective setting. To fully assess the clinical applicability, future studies should investigate how these maps perform in a prospective clinical workflow for diagnosis. We plan to conduct a study that will include evaluating how radiologists interact with AI-ADC maps in real-time, and whether these improvements in image quality translate into better diagnostic outcomes, such as more accurate detection of clinically significant prostate cancer or reduction in unnecessary biopsies. Since the primary goal of the study was not the oncologic outcome, we did not explore this important point in sufficient detail. Finally, generative AI carries the risk of hallucinations; while we have not observed this in our initial development study and current work, a technical safety analysis in a large-scale diagnostic study is among our plans in the near future.

In conclusion, this study demonstrates the feasibility of AI-ADC maps in replacing standard ADC maps. AI-ADC maps have enhanced quality, improved delineation of prostate boundaries, reduced noise, and minimized distortion. These improvements can streamline diagnostics by reducing the number of initial acquisitions and the need for reacquisitions. However, further testing is necessary to determine whether AI-ADC will lead to faster scans with better clinical decision-making.

## Supplementary information

ELECTRONIC SUPPLEMENTARY MATERIAL

## Abbreviations

ADC: Apparent diffusion coefficient
AI: Artificial intelligence
AI-ADC: Artificial intelligence-generated apparent diffusion coefficient
CI: Confidence interval
CLMM: Cumulative link mixed model
csPCa: Clinically significant prostate cancer
DW MRI: Diffusion-weighted magnetic resonance imaging
DWI: Diffusion-weighted imaging
mpMRI: Multiparametric magnetic resonance imaging
MRI: Magnetic resonance imaging
PCa: Prostate cancer
PI-QUAL: Prostate imaging quality
PI-RADSv2.1: Prostate Imaging Reporting and Data System
PSA: Prostate-specific antigen
QWK: Quadratic-weighted kappa
T2W MRI: T2-weighted magnetic resonance imaging
