# Supplementary material for: Artificial intelligence–generated apparent diffusion coefficient (AI-ADC) maps for prostate gland assessment: a multi-reader study
Source: Eur Radiol. 2025 Jul 21;36(1):288–95. doi: 10.1007/s00330-025-11871-z (PMC12712018; doi:10.1007/s00330-025-11871-z)
Supplement: Supplementary file 1 — ELECTRONIC SUPPLEMENTARY MATERIAL [file 330_2025_11871_MOESM1_ESM.pdf]

Artificial Intelligence–Generated Apparent Diffusion Coefficient (AI-ADC) Maps for Prostate Gland Assessment: A Multi-Reader Study

ELECTRONIC SUPPLEMENTARY MATERIAL

Graphical User Interface for Image Evaluation Rounds

For consistency during image evaluations, we utilized a customized web interface. In compliance with HIPAA, all patient images were fully deidentified prior to release to our readers and uploaded to a secure website for viewing and assessing the scans. When readers click on the ‘View Images’ button they visualized, side-by-side cropped T2W-MRI and ADC maps (either standard ADC or AI-ADC maps) as shown in Figure 2. The readers could synchronously zoom both T2W MRI and ADC maps. They had the ability to adjust the windowing settings and could reset the view if they wished to start over with different viewing options. We presented imaging pairs in a scrambled fashion and ensured that the readers did not view the same patient’s real and generated ADC maps in the same evaluation round. However, it was possible for readers to observe both ADC and AI-ADC maps from different patients within the same round.

Reader Study Questions

I. Synchronization: Please make sure the “Stack Image Sync ” button from the top toolbar is selected. After reviewing the provided axial T2-weighted imaging (T2WI) and the corresponding Apparent Diffusion Coefficient (ADC) map, please indicate whether both images are aligned in the same axial orientation using the internal and external structures such as prostatic capsule, anterior fibromuscular stroma, urethra, seminal vesicles.

a. Yes, they are aligned.
b. No, they are not aligned.
c. Not possible to tell.

II. Motion (Z-axis): Please indicate whether there is motion along the Z-axis on the T2WI and ADC map?

a. Yes.
b. No.

III. Windowing: Please indicate how easy it was to window the provided ADC map for your interpretation.

a. Did not need to window.
b. Easy
c. Moderate
d. Difficult

IV. Boundaries: Please evaluate the ADC map for the delineation of the outer prostate boundary and differentiation of the peripheral zone from the transition zone using the following 4-point scale?

a. Excellent
b. Adequate
c. Marginally acceptable
d. Not acceptable

V. Distortion: Could you please evaluate the ADC map for presence of distortion?

a. None or minimal, there is no impact on interpretation.
b. Mild, there is minimal impact on interpretation.
c. Moderate, interpretation is hampered.
d. Severe, interpretation is totally precluded.

VI. Distortion - Location: Could you please indicate the prostatic segments impacted by distortion on the ADC map?

a. N/A, there is no distortion.
b. Apex
c. Apical-mid
d. Apical-base
e. Mid-gland
f. Mid-base
g. Base

VII. Noise: Could you please evaluate the ADC map for presence of noise?

a. None or minimal, there is no impact on interpretation.
b. Mild, there is minimal impact on interpretation.
c. Moderate, interpretation is hampered.
d. Severe, interpretation is totally precluded.

VIII. Artifact: Could you please evaluate the ADC map for presence of implant/instrument-related artifact?

a. None or minimal, there is no impact on interpretation.
b. Mild, there is minimal impact on interpretation.
c. Moderate, interpretation is hampered.
d. Severe, interpretation is totally precluded.

IX. Artifact - Location: Could you please indicate the prostatic segments impacted by implant/instrument-related artifacts on the ADC map?

a. N/A, there is no distortion.

b. Apex
c. Apical-mid
d. Apical-base
e. Mid-gland
f. Mid-base
g. Base

X. Overall QC – ADC map: How would you categorize the overall “ADC map” quality?
a. Low quality
b. High quality

XI. Repeat – ADC map: Would you prefer to repeat the image acquisition for the ADC map?
a. Yes
b. No

XII. Overall QC – T2WI: How would you categorize the overall “T2WI” quality?
a. Low quality
b. High quality

Supplementary Table 1: T2W MRI and ADC Maps Acquisition Parameters.

Note: ADC = Apparent diffusion coefficient calculation was based on three evenly spaced acquired b-values (0-600 s/mm^2). (T2W MRI = T2-weighted Magnetic Resonance Imaging)

Parameters	T2W MRI	ADC Maps
**Field of view (mm)**	180 × 180	140 × 140
**Acquisition matrix**	220 × 207	80 × 80
**Repetition time (ms)**	3644	5102
**Echo time (ms)**	110	77
**Flip angle (degrees)**	90	90
**Section thickness (mm)**	3	3
**Image reconstruction matrix (pixels)**	512 × 512	144 × 144
**Reconstruction voxel imaging resolution (mm/pixel)**	0.31 × 0.31 × 3	1.11 × 1.11 × 3
**Echo train length**	20	71
**Number of averages**	2	3

Supplementary Table 2: Summary of Reader Responses for Qualitative Comparison of ADC and AI-ADC maps. R1 = Reader 1, R2 = Reader 2, R3 = Reader 3, R4 = Reader 4.

Criteria	Reader	AI-ADC > ADC	AI-ADC = ADC	ADC > AI-ADC	Wald-Stat [95 % CI]	*p*-value
**Windowing Easiness**	R1	21.62% (16)	60.81% (45)	17.57% (13)	[-0.108, 0.189]	$p > 0.05$
	R2	51.35% (38)	32.43% (24)	16.22% (12)	[0.176, 0.527] 0.001	$p <$
	R3	0% (0)	97.30% (72)	2.70% (2)	[-0.068, 0.000]	$p > 0.05$
	R4	37.84% (28)	36.49% (27)	25.68% (19)	[-0.068, 0.284]	$p > 0.05$
**Prostate Boundary Delineation**	R1	28.38% (21)	48.65% (36)	22.97% (17)	[0.122, 0.216]	$p > 0.05$
	R2	45.95% (34)	40.54% (30)	13.51% (10)	[0.149, 0.487]	$p < 0.001$
	R3	62.16% (46)	27.03% (20)	10.81% (8)	[0.338, 0.662]	$p < 0.001$
	R4	36.49 % (27)	54.05% (40)	9.46% (7)	[0.108, 0.405]	$p < 0.001$
**Distortion Evaluation**	R1	25.68% (19)	63.51% (47)	10.81% (8)	[0.014, 0.270]	$p < 0.05$
	R2	43.24% (32)	50.00% (37)	6.76% (5)	[0.216, 0.487]	$p < 0.001$
	R3	58.11% (43)	25.68% (19)	16.22% (12)	[0.230, 0.595]	$p < 0.001$
	R4	52.70% (39)	35.14% (26)	12.16% (9)	[0.243, 0.568]	$p < 0.001$
**Noise Evaluation**	R1	20.27% (15)	56.76% (42)	22.97% (17)	[-0.176, 0.135]	$p > 0.05$
	R2	40.54% (30)	51.35% (38)	8.11% (6)	[0.189, 0.446]	$p < 0.001$
	R3	43.24% (32)	44.59% (33)	12.16% (9)	[0.149, 0.446]	$p < 0.001$
	R4	16.22% (12)	66.22% (49)	17.57% (13)	[-0.135, 0.135]	$p < 0.001$
**Overall Quality Evaluation of T2W MRI**	R1	2.70% (2)	93.74% (69)	4.05% (3)	[-0.068,0.041]	$p > 0.05$
	R2	14.86% (11)	77.03% (57)	8.11% (6)	[-0.041, 0.189]	$p > 0.05$

	R3	5.41% (4)	86.49% (64)	8.11% (6)	[-0.122, 0.054]	*p* > 0.05
	R4	6.76% (5)	90.54% (67)	2.70% (2)	[-0.027, 0.108]	*p* > 0.05
**Overall Quality Evaluation of ADC Maps**	R1	21.62% (16)	72.97% (54)	5.41% (4)	[0.054, 0.270]	*p* < 0.01
	R2	39.19% (29)	56.76% (42)	4.05% (3)	[0.230, 0.473]	*p* < 0.001
	R3	37.84% (28)	55.41% (41)	6.76% (5)	[0.176, 0.446]	*p* < 0.001
	R4	40.54% (30)	55.41% (41)	4.05% (3)	[0.230, 0.487]	*p* < 0.001
**Image Reacquisition Requirements for ADC Maps**	R1	20.27% (15)	74.32% (55)	5.41% (4)	[0.040, 0.257]	*p* < 0.01
	R2	37.84% (28)	58.11% (43)	4.05% (3)	[0.216, 0.473]	*p* < 0.001
	R3	18.92% (14)	78.38% (58)	2.70% (2)	[0.068, 0.257]	*p* < 0.01
	R4	41.89% (31)	54.05% (40)	4.05% (3)	[0.243, 0.500]	*p* < 0.001

Supplementary Table 3: Summary of Reader Responses for Ordinary Scoring Comparison of ADC and AI-ADC maps' Scores. R1 = Reader 1, R2 = Reader 2, R3 = Reader 3, and R4 = Reader 4.

Questions	Modality\Readers	R1	R2	R3	R4
**Windowing Easiness (/4)**	ADC maps	2.26 ± 0.44	1.12± 0.89	3.00±0.00	1.61±0.79
	AI-ADC maps	2.30 ± 0.46	1.64±0.79	2.95±0.00	1.81±0.79
	Wilcox-p	$p > 0.05$	$p < 0.001$	$p > 0.05$	$p > 0.05$
**Prostate Boundary Delineation**	ADC maps	1.96 ± 0.73	1.35±0.94	1.36 ±0.73	1.84±0.66
	AI-ADC maps	2.07 ± 0.69	1.82 ± 0.91	2.12 ± 0.83	2.19±0.61
	Wilcox-p	p >0.05	$p < 0.001$	$p < 0.001$	$p < 0.001$
**Distortion Level (/4)**	ADC maps	1.96 ± 0.73	1.35±0.94	1.36 ±0.73	1.88±0.89
	AI-ADC maps	2.07 ± 0.69	1.82 ± 0.91	2.12 ± 0.83	2.43 ± 0.64
	Wilcox-p	$p < 0.05$	$p < 0.001$	$p < 0.001$	$p < 0.001$
**Noise Level (/4)**	ADC maps	1.51 ± 0.69	2.08 ± 1.06	1.54 ± 0.65	1.23 ± 0.45
	AI-ADC maps	1.53 ± 0.83	2.59 ± 0.83	2.03 ± 0.84	1.32 ± 0.72
	Wilcox-p	$p > 0.05$	$p < 0.001$	$p < 0.001$	$p > 0.05$
**ADC Maps Overall Quality (/2)**	ADC maps	0.69 ± 0.47	0.41 ± 0.49	0.43 ± 0.50	0.53 ± 0.50
	AI-ADC maps	0.85 ± 0.36	0.76 ± 0.43	0.74 ± 0.44	0.89 ± 0.31
	Wilcox stat	$p < 0.05$	$p < 0.001$	$p < 0.001$	$p < 0.001$
**T2W MRI Quality (/2)**	ADC maps	0.91 ± 0.29	0.58 ± 0.50	0.86 ±0.34	0.89 ±0.31
	AI-ADC maps	0.89 ± 0.31	0.65 ± 0.48	0.84 ± 0.37	0.93 ± 0.25
	Wilcox-p	$p > 0.05$	$p > 0.05$	$p > 0.05$	$p > 0.05$
**Reacquisition Requirement (/2)**	ADC maps	0.70 ± 0.46	0.39 ± 0.37	0.80 ± 0.40	0.53 ± 0.50
	AI-ADC maps	0.85 ± 0.36	0.73 ± 0.45	0.96 ± 0.20	0.91 ± 0.29
	Wilcox-p	$p < 0.05$	$p < 0.001$	$p < 0.001$	$p < 0.001$

Supplementary Table 4: Inter-reader Agreement Bootstrap Analysis for Quadratic Weighted Kappa. (95% confidence intervals are calculated from bootstrap estimates) R1 = Reader 1, R2 = Reader 2, R3 = Reader 3, and R4 = Reader 4. QWK_{ADC} = Quadratic Weighted Kappa on ADC Maps scores, QWK_{AI-ADC} = Quadratic Weighted Kappa on AI-ADC Maps scores, and QWK_{diff} = Quadratic Weighted Kappa on the score difference of ADC and AI-ADC maps.

		Reader Pairs					
Criteria	Metric	R1 R2	R1 R3	R1 R4	R2 R3	R2 R4	R3 R4
**Windowing Easiness**	QWK_{ADC}	-0.131 [-0.289,0.023]	0 [0.000-0.000]	0.096 [-0.096,0.291]	0 [0.000-0.000]	-0.012 [-0.221,0.213]	0 [0.000,0.000]
	QWK_{AI-ADC}	0.243 [0.050,0.430]	0.043 [0.000,0.111]	-0.050 [-0.227,0.150]	0.002 [-0.054,0.049]	-0.004 [-0.182,0.180]	-0.031 [-0.104,0.007]
	QWK_{diff}	0.254 [0.055,0.397]	0.009 [-0.024,0.043]	0.007 [-0.193,0.197]	0.001 [-0.060,0.060]	0.224 [0.019,0.413]	-0.107 [-0.282,0.027]
**Prostate Boundary Delineation**	QWK_{ADC}	0.292 [0.119,0.457]	0.185 [-0.003,0.375]	0.396 [0.159,0.598]	0.377 [0.175,0.544]	0.268 [0.084,0.430]	0.203 [0.033,0.369]
	QWK_{AI-ADC}	0.479 [0.243,0.657]	0.512 [0.273,0.712]	0.315 [-0.014,0.578]	0.279 [0.009,0.504]	0.054 [-0.192,0.301]	0.221 [-0.092,0.491]
	QWK_{diff}	0.364 [0.140,0.483]	0.316 [0.122,0.401]	0.310 [-0.000,0.391]	0.222 [-0.067,0.330]	0.327 [0.038,0.403]	0.207 [0.028,0.385]
**Distortion Evaluation**	QWK_{ADC}	0.078 [-0.156,0.300]	-0.104 [-0.307,0.111]	0.198 [-0.006,0.401]	0.021 [-0.177,0.220]	0.184 [0.008,0.353]	-0.126 [-0.349,0.099]
	QWK_{AI-ADC}	0.2474 [0.039,0.460]	0.390 [0.180,0.608]	0.0148 [-0.181,0.215]	0.285 [0.104,0.478]	0.113 [-0.042,0.277]	-0.001 [-0.197,0.221]
	QWK_{diff}	0.3701 [0.076,0.417]	0.181 [-0.008,0.287]	0.317 [0.091,0.377]	0.418 [0.172,0.477]	0.492 [0.227,0.484]	0.182 [-0.051,0.350]
**Noise Evaluation**	QWK_{ADC}	0.062 [-0.042,0.164]	-0.045 [-0.253,0.162]	-0.026 [-0.157,0.152]	-0.079 [-0.188,0.032]	0.003 [-0.069,0.067]	-0.001 [-0.167,0.177]
	QWK_{AI-ADC}	0.015 [-0.087,0.106]	0.163 [-0.062,0.367]	0.207 [-0.022,0.416]	0.024 [-0.086,0.154]	0.034 [-0.016,0.090]	0.069 [-0.099,0.237]
	QWK_{diff}	-0.190 [-0.354, -0.063]	0.084 [-0.160,0.218]	0.182 [-0.034,0.349]	-0.043 [-0.220,0.140]	-0.020 [-0.289,0.062]	-0.034 [-0.220,0.176]
**Overall Quality Evaluation of T2W MRI**	QWK_{ADC}	0.253 [0.105,0.423]	0.404 [0.095,0.687]	0.333 [-0.057,0.648]	0.356 [0.191,0.541]	0.288 [0.109,0.480]	0.495 [0.164, 0.786]
	QWK_{AI-ADC}	0.366 [0.181,0.565]	0.426 [0.098,0.702]	0.077 [-0.112,0.370]	0.256 [0.049,0.471]	0.163 [0.002,0.347]	0.415 [0.097, 0.687]
	QWK_{diff}	0.097 [-0.010,0.267]	-0.004 [-0.349,0.348]	-0.324 [-0.637,0.026]	0.157 [0.000,0.329]	-0.017 [-0.072,0.018]	0.009 [-0.308, 0.299]
**Overall Quality Evaluation of ADC Maps**	QWK_{ADC}	0.420 [0.255,0.587]	0.306 [0.121, 0.485]	0.448 [0.237-0.633]	0.501 [0.297,0.690]	0.492 [0.286, 0.674]	0.490 [0.286, 0.676]
	QWK_{AI-ADC}	0.535 [0.269,0.752]	0.425 [0.176-0.651]	0.338 [0.026-0.629]	0.532 [0.293,0.734]	0.367 [0.113, 0.607]	0.432 [0.197, 0.669]
	QWK_{diff}	0.311 [0.112,0.503]	0.319 [0.120-0.493]	0.299 [0.104-0.477]	0.447 [0.241,0.626]	0.327 [0.124, 0.486]	0.390 [0.197, 0.555]
**Image Reacquisition Requirements for ADC Maps**	QWK_{ADC}	0.379 [0.210,0.559]	0.252 [0.002,0.464]	0.475 [0.281,0.655]	0.234 [0.090,0.385]	0.519 [0.324,0.699]	0.329 [0.151, 0.528]
	QWK_{AI-ADC}	0.481 [0.230,0.710]	-0.068 [-0.128,0.000]	0.246 [-0.057,0.544]	0.111 [-0.051,0.310]	0.268 [0.054,0.498]	0.364 [0.098, 0.236]
	QWK_{diff}	0.248 [0.056,0.434]	0.141 [-0.107,0.372]	0.306 [0.126,0.470]	0.049 [-0.126,0.207]	0.284 [0.080,0.447]	0.267 [0.043,0.483]

	Fleiss' κ for ADC Maps	Fleiss' κ for AI-ADC Maps	Fleiss' κ for ADC and AI-ADC Maps Scoring Difference
**Windowing Easiness**	-0.087<br>[-0.104, -0.073]	-0.076<br>[-0.098, -0.055]	-0.031<br>[-0.085, 0.022]
**Prostate Boundary Delineation**	0.04<br>[-0.001, 0.075]	0.081<br>[0.027, 0.128]	0.079<br>[0.005, 0.137]
**Distortion Evaluation**	0.081<br>[0.043, 0.113]	0.048<br>[-0.005, 0.094]	0.085<br>[0.023, 0.140]
**Noise Evaluation**	0.009<br>[-0.032, 0.042]	0.003<br>[-0.039, 0.038]	0.042<br>[-0.027, 0.106]
**Overall Quality Evaluation of T2W MRI**	0.052<br>[-0.017, 0.113]	0.030<br>[-0.030, 0.084]	0.023<br>[-0.053, 0.092]
**Overall Quality Evaluation of ADC Maps**	0.160<br>[0.097, 0.215]	0.096<br>[0.008, 0.167]	0.278<br>[0.155, 0.387]
**Image Reacquisition Requirements for ADC Maps**	0.117<br>[0.06, 0.168]	0.005<br>[-0.05, 0.046]	0.150<br>[0.044, 0.245]

Supplementary Table 5: Inter-reader Agreement Bootstrap Analysis for Fleiss' Kappa (κ). (95% confidence intervals are calculated from bootstrap estimates.)

Supplementary Figure

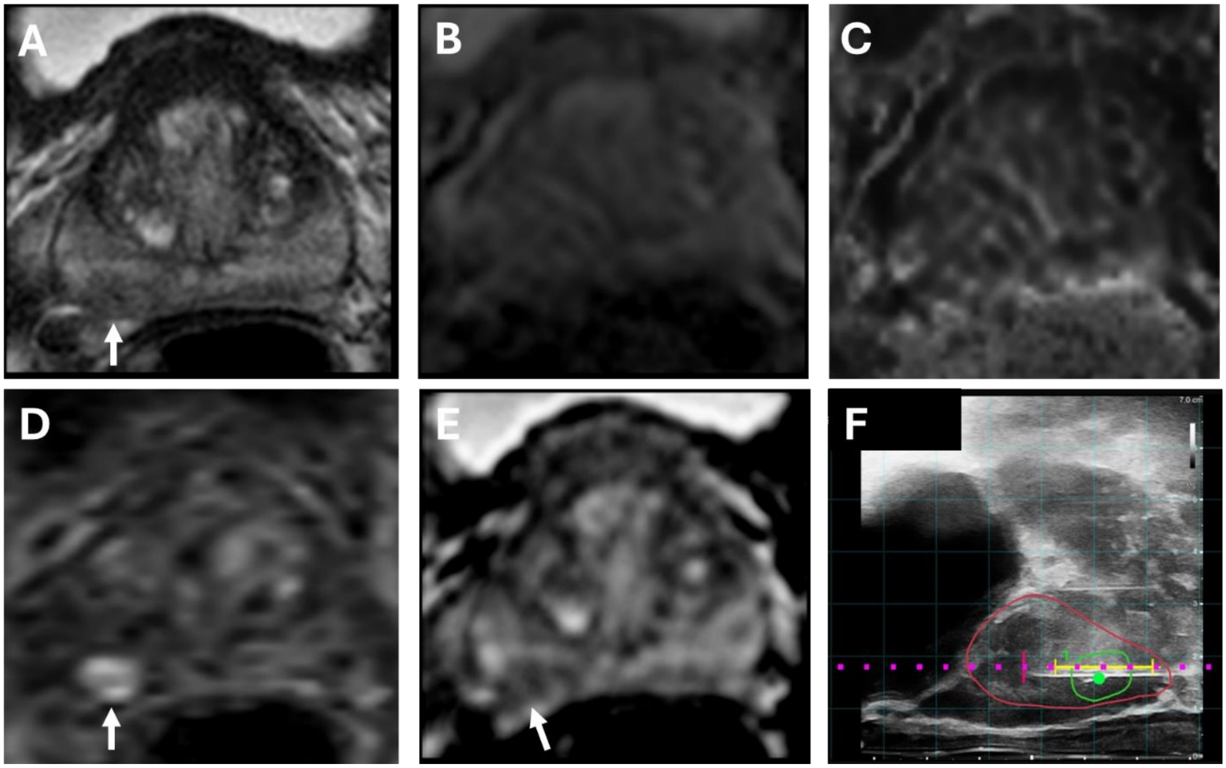

Supplementary Figure 1: 69-year-old male with a serum PSA of 3.3ng/ml. Axial T2W MRI shows a PI-RADS 3 lesion in the right mid peripheral zone (arrow) (A), which is invisible on ADC map (B) and calculated b=1500 DW MRI (C); the lesion demonstrates early focal enhancement on DCE MRI (arrow) (D). The right mid peripheral zone lesion appears hypointense on the AI-ADC map (arrow) (E). TRUS/MRI fusion guided biopsy via transperineal approach revealed Gleason 3+4 prostate cancer with porrly formed glands and perineural invasion (F).
